# Early ovariectomy reveals the germline encoding of natural anti‐A‐ and Tn‐cross‐reactive immunoglobulin M (IgM) arising from developmental *O*‐GalNAc glycosylations. (Germline‐encoded natural anti‐A/Tn cross‐reactive IgM)

**DOI:** 10.1002/cam4.1079

**Published:** 2017-06-05

**Authors:** Peter Arend

**Affiliations:** ^1^ Philipps University Marburg Department of Medicine D‐355 Marburg/Lahn, Germany; ^2^ Gastroenterology Research Laboratory University of Iowa, College of Medicine Iowa City Iowa; ^3^ Research Laboratories Chemie Grünenthal GmbH D‐52062 Aachen Germany

**Keywords:** Developmental location, glycosidic accommodation, *Helix pomatia* reactivity, nonimmune IgM, invertebrate defense proteins, non‐developmental tissue, *O*‐GalNAc glycosylation(s)

## Abstract

While native blood group A‐like glycans have not been demonstrated in prokaryotic microorganisms as a source of human “natural” anti‐A isoagglutinin production, and metazoan eukaryotic *N*‐acetylgalactosamine *O*‐glycosylation of serine or threonine residues (*O*‐GalNAc‐Ser/Thr‐R) does not occur in bacteria, the *O*‐GalNAc glycan‐bearing ovarian glycolipids, discovered in C57BL/10 mice, are complementary to the syngeneic anti‐A‐reactive immunoglobulin M (IgM), which is not present in animals that have undergone ovariectomy prior to the onset of puberty. These mammalian ovarian glycolipids are complementary also to the anti‐A/Tn cross‐reactive *Helix pomatia* agglutinin (HPA), a molluscan defense protein, emerging from the coat proteins of fertilized eggs and reflecting the snail‐intrinsic, reversible *O*‐GalNAc glycosylations. The hexameric structure of this primitive invertebrate defense protein gives rise to speculation regarding an evolutionary relationship to the mammalian nonimmune, anti‐A‐reactive immunoglobulin M (IgM) molecule. Hypothetically, this molecule obtains its complementarity from the first step of protein glycosylations, initiated by GalNAc via reversible *O*‐linkages to peptides displaying Ser/Thr motifs, whereas the subsequent transferase depletion completes germ cell maturation and cell renewal, associated with loss of glycosidic bonds and release of *O*‐glycan‐depleted proteins, such as complementary IgM revealing the structure of the volatilely expressed “lost” glycan carrier through germline Ser residues. Consequently, the evolutionary/developmental first glycosylations of proteins appear metabolically related or identical to that of the mucin‐type, potentially “aberrant” monosaccharide GalNAc*α*1‐*O*‐Ser/Thr‐R, also referred to as the Tn (T “nouvelle”) antigen, and explain the anti‐Tn cross‐reactivity of human innate or “natural” anti‐A‐specific isoagglutinin and the pronounced occurrence of cross‐reactive anti‐Tn antibody in plasma from humans with histo‐blood group O. In fact, A‐allelic, phenotype‐specific GalNAc glycosylation of plasma proteins does not occur in human blood group O, affecting anti‐Tn antibody levels, which may function as a growth regulator that contributes to a potential survival advantage of this group in the overall risk of developing cancer when compared with non‐O blood groups.

## Introduction

While the naturally occurring immunoglobulin M (IgM) is permanently engaged in recognition and elimination of aberrant growth and cancerous tissue, the secretion of IgM molecules is not restricted to B cells but spontaneously occurs in murine 1, 2 and human 3 normal and malignant epithelial cells as well. Moreover, although many anti‐glycan antibodies do not adhere to the paradigm of an adaptive immune response and are often referred to as “natural antibodies” 4, to date, based on the historical experiments of Springer et al. 5, 6, 7, the production of human histo‐blood group ABO(H) isoantibodies or isoagglutinins with Tn and T antigen cross‐specificity is believed to be exclusively induced by environmental, predominantly intestinal, well‐documented microbial antigens, particularly lipopolysaccharides from gram‐negative bacteria. However, prokaryotic “blood group A/B‐like” antigenic structures basically induce cross‐reactive anti‐A/B immunoglobulins, which due to clonal selection neither arise in blood group A nor in B individuals. While bacterial endotoxins nonspecifically stimulate the formation of all immunoglobulins, most likely involving the anti‐A/B isoagglutinins, a definitive adaptive, enteral immunization with ABO(H)‐reactive, environmental antigens is a source of antibody production that in humans might largely be restricted to blood group O(H) individuals. When adaptive production of anti‐blood group B‐reactive immunoglobulins occurring in White Leghorn chickens fed a diet containing E.coli O86:B7 lipopolysaccharide 5, was demonstrated for the first time to occur spontaneously in humans 8, this way of isoagglutinin production could exclusively be documented for the histo (blood) group O(H). Although this blood group can no longer be considered a genetic entity, which in particular is contaminated by OA hybrid or weak A alleles 9, 10, even a small number of blood group O(H) patients, suffering from ulcerative colitis associated with increased enteral absorption, showed a statistically significant adaptive immune response, measured by an anti‐B‐reactive 7S (IgG)‐ and 19S (IgM) immunoglobulin, involving asymetrically cross‐reactive, less pronounced anti‐A‐reactive IgG, whereas the anti‐B‐reactive IgG and IgM antibody levels in plasma from blood group A patients remained within normal range 11.

Chemical immunosuppressants, which are used prior to major ABO‐incompatible transplantations to downregulate immunoglobulin synthesis by the recipient, do not completely eliminate the anti‐A and anti‐B reactivity of different immunoglobulin classes 12. In fact, chemical immunosuppression does not affect the formation of the mercaptoethanol‐sensitive, complement‐binding anti‐A/B “classic” isoagglutinins that preferentially induce hemagglutination at 22–24°C. These hemagglutinins must be removed via plasmapheresis or specific adsorption.

**Figure 1:**
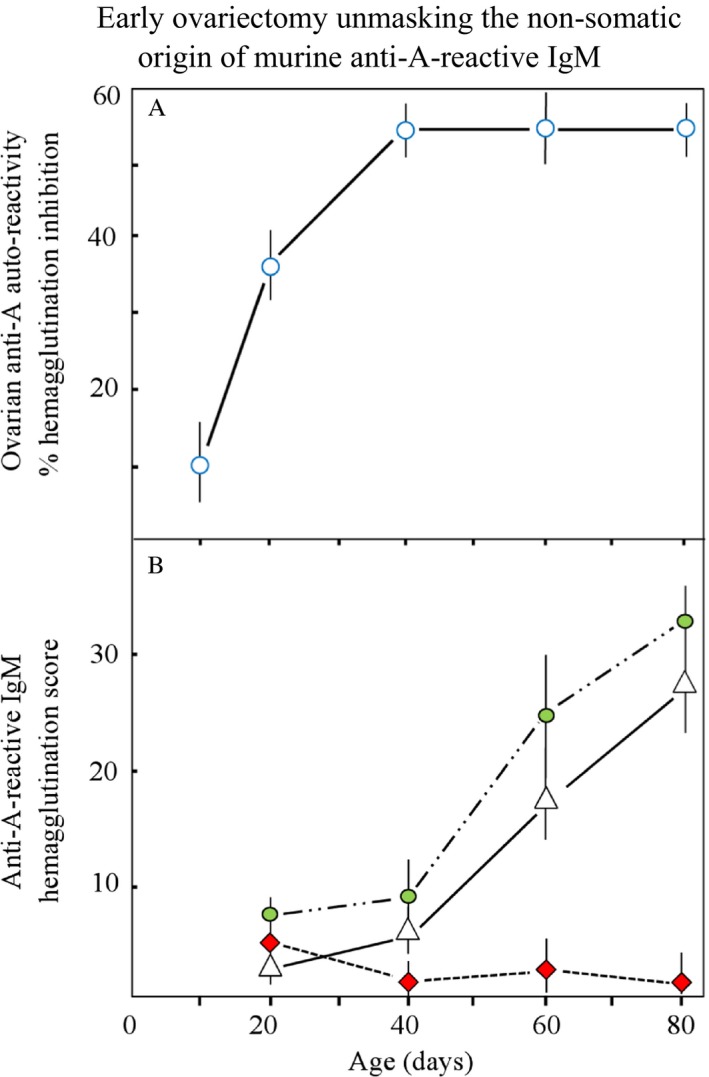
(A) Growth‐related appearance of autoreactive, nonsomatic GalNAc glycan–bearing hydrophilic glycolipids in differentiating ovarian tissue, with peak levels appearing at puberty. (B) Subsequently arising complementary, innate anti‐A reactive IgM: Serum of untreated animals (white triangles), sham‐operated (green ovals), ovariectomized (red squares). This development of innate anti‐A reactivity does not reflect (auto) immune response but signifies the completion of cell differentiations and shows IgM release during deglycosylations. Figure reconstructed from Arend and Nijssen (1977, *Nature*, 269, 255–257) 16, cited in Arend (2016, ABO (histo) blood group phenotype development and human reproduction as they relate to ancestral IgM formation: A hypothesis. *Immunobiology*, 221(1), 116–127, PMID: 26433867) [80].

Neither *N*‐ nor *O*‐linked native blood group A‐like glycans have been demonstrated in prokaryotic microorganisms; in particular, mucin‐type GalNAc‐*O*‐Ser/Thr glycosylation does not occur in bacteria 13. The synthesis of *O*‐linked GalNAc glycan‐bearing ovarian glycolipids, discovered in C57BL/10 mice, is associated with the formation of a syngeneic, complementary anti A‐reactive IgM 14, 15, which demonstrates identical serological reaction patterns to human innate anti‐A isoagglutinin but is not present in animals that have undergone ovariectomy prior to the onset of puberty 15, 16 (Fig. 1). Furthermore, the anti‐A/B cross‐reactive antibody produced when White Leghorn chickens were fed a diet containing *Escherichia coli* O86:B7 lipopolysaccharide 5, appeared identically in C57BL/10 mice when immunized with the same antigen; however, this immunization did not affect pre‐existing levels of the syngeneic “natural” anti‐A antibody, which was simply separated from the adaptive, cross‐reactive antibody via specific adsorption 17.

**Figure 2:**
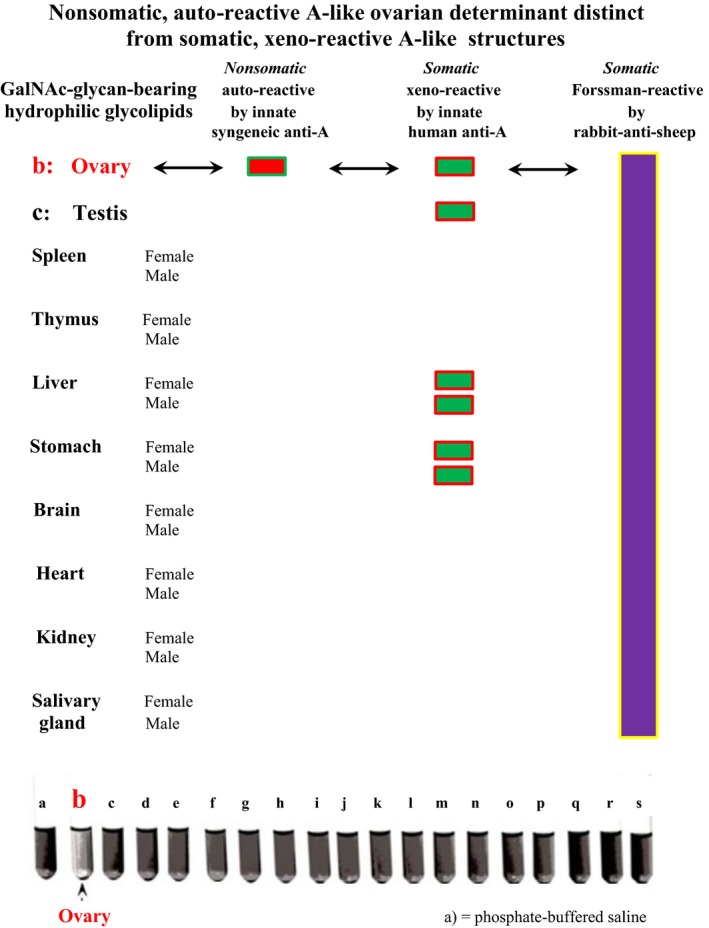
Distribution of autoreactive/nonsomatic and xenoreactive/somatic GalNAc glycan‐bearing hydrophilic glycolipids in C57BL/10 murine ovarian and nonreproductive tissues. While all the murine tissues exhibit characteristic species‐intrinsic Forssman reactivity and other xenoreactive A‐like structures in male and female reproductive, and endodermal organs are detected using innate human anti‐A antibodies, the murine anti‐A antibody was exclusively inhibited by syngeneic ovarian glycolipids. The image was captured during the hemolysis inhibition experiments described in Arend (1980, *Immunobiology*, 156, 410–417) 18, cited in Arend (2016, *Immunobiology*, 219, 285–29, PMID: 26433867) 80.

All murine tissues expressed the expected species‐intrinsic Forssman‐type structure, and additional A‐like structures in the male and female reproductive organs and endodermal tissues were detected using human anti‐A antibody, whereas the murine anti‐A molecule was exclusively inhibited by syngeneic ovarian glycolipids 18 (Fig. 2). These crude glycolipid preparations showed developmental polymorphism, which was identified through reactions with *Dolichos biflorus* lectin and *Helix pomatia* agglutinin (HPA) 16, revealing the involvement of mucin‐type *O*‐GalNAc‐determined serologically “A‐like” glycans in complex protein glycosylation processes, such as characterizing T‐cell development. A major cell surface glycoprotein (apparent mol. wt. = 150,000) on human lymphocytes has been reported to provide HPA‐binding or the presence of HPA receptor activity on normal and malignant thymus‐derived (T) lymphocytes 19. Such binding was not found on various B cells at different steps of differentiation, whereas two of four B cell lymphoma lines and a myeloma line had another HPA‐binding surface glycoprotein (mol. wt. = 200,000) instead of the 150,000–mol. wt. protein. The serologically “A‐like” HPA receptor, or mucin‐type GalNAc*α*1‐*O*‐Ser/Thr‐R glycan, also referred to as the Tn antigen 20, has been reported as a surface marker on natural killer cells (NK) in normal mouse spleen after neuraminidase treatment 21, 22, while its expression appears to be dependent on the level of Ser/Thr‐specific protein kinase C 23. This enzyme obviously activates to the family of those glycotransferases, providing the first step of protein glycosylation that in metazoan eukaryotes is initiated by GalNAc via *O*‐linkages 24, 25, and is essential in T cell activation and downregulation, performed through macrophage galactose lectin (MGL), also termed “Tn lectin”.

Not spontaneously occurring in plant species [13], the Tn antigen is a common metazoan eukaryotic structure, which arises from *O*‐GalNAc glycosylations, used already by mollusks and insects, like the fruit fly *Drosophila melanogaster* 26 but when arrested in nondevelopmental tissues of higher metazoans, such as mammals, signifies malignancy, while the degree of HPA binding correlates with the stages of various kinds of metastatic cancer, irrespectively of the organ 27, 28, 29, 30. Furthermore, when animal tumors are associated with Tn antigen expression 31, it is important to mention that the HPA binding sites are identical to that in human tumors and appear to change similarly with tumor stage. The histochemistry of murine WAP‐T mammary cancer has revealed glycoconjugate changes similar to that in human breast cancer 32. In plasma, the major HPA‐binding proteins are blood group ABO(H)‐reactive glycoproteins, such as clotting factor VIII (FVIII)^33^ and von Willebrand factor (vWF) 34, carried by *α*2‐macroglobulin (A2M) 35. This is an abundant polyfunctional protein occurring in plasma of mammals and considered an evolutionarily conserved arm of the innate immune system 36, while in the human is expressing the ABO(H) phenotype in plasma, strictly in accordance with the expression on red cell surfaces 35. Thus, when using HPA for identifying cancer biomarkers in sera and plasma 28, determining ABO(H) phenotype is of utmost importance.

## Cross‐specificity between mammalian anti‐A/anti‐Tn/T‐reactive IgM and invertebrate defense proteins reveals the evolutionary/developmental position of Tn/T epitopes

**Figure 3:**
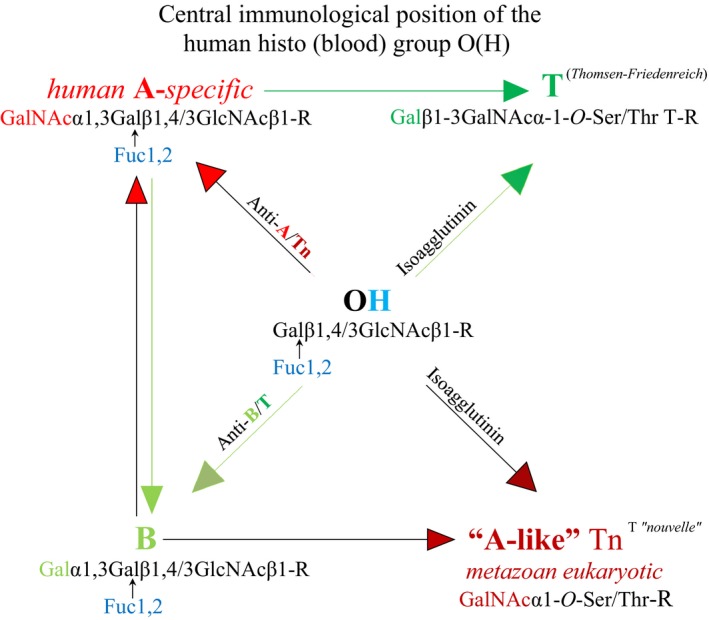
The central immunological position of blood group O(H) is evident in its comprehensive presentation of adaptive and innate “natural” antibodies against all mature A and B glycans and their cross‐reactive developmental structures Tn and T. The human A‐specific (A‐allelic) glycosylation and trans‐species “A‐like” Tn formation are developmentally connected via the formation of cross‐reactive anti‐A/Tn isoagglutinin. According to Hofmann et al. 57, blood O(H) sera bind to both Tn and T antigens, and the anti‐A isoagglutinin levels in blood group O(H) and blood group B sera are associated with anti‐Tn antibody, which does not react with blood group B red cells or T glycoconjugates. In contrast, the anti‐B antibodies of blood group A sera and blood group O(H) sera bind to B and T glycoconjugates but not to A or Tn glycoconjugates. The authors explain this selective cross‐reactivity of isoagglutinins with Tn and T antigens via phenotype‐specific terminal moieties; the terminal *N*‐acetylgalactosamine is shared by A and Tn antigens, and the terminal galactose is, although with different configuration, shared by B and T antigens.

**Figure 4:**
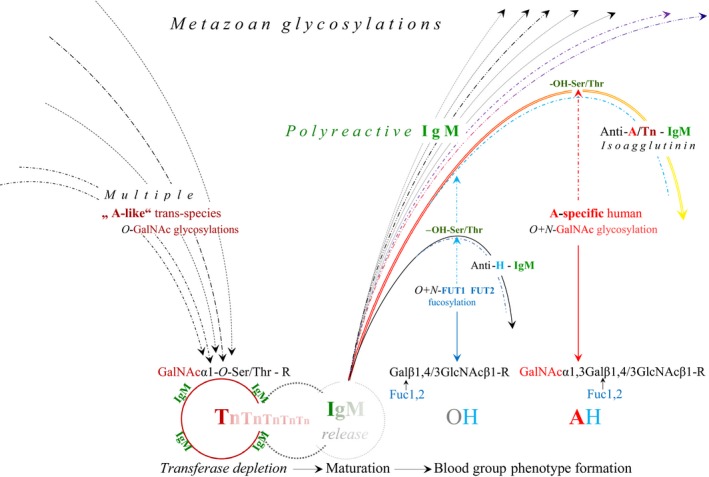
Hypothetical germline encoding of nonimmune, natural anti‐A/cross‐reactive anti‐Tn IgM and phenotype‐specific, glyosidic accommodation of plasma proteins. The *metazoan* trans‐species “A‐like” *O*‐GalNAc glycosylations of proteins, involving the formation of the mucin‐type *O*‐glycan, GalNAc*α*1‐*O*‐Ser/Thr‐R, also referred to as the Tn antigen, are distinct from the species‐specific *N+O*‐linked GalNAc glycosylation or blood group A phenotype formation. The naturally occurring anti‐A isoagglutinin and the anti‐Tn reactivity in human plasma cannot be separated from each other; they are expressed together by a secretory, primarily polyreactive IgM molecule, which arises in connection with the transient expression of the Tn antigen during the course of ESC‐GC transformation and cell renewal processes.The in normal conditions extremely short half‐live of this expression concerns the binding of the nonimmune IgM molecule, which has developed as a cell adhesion molecule and is released after Tn depletion. This secretory IgM molecule retains the Ser/Thr‐OH functional groups that in the normal human undergo the ABOH) phenotype formation, occurring on both cell surfaces and plasma proteins, and involves *N‐* and *O*‐glycosylations. This process is based on human‐specific FUT1 and FUT2 fucosylations, which exclude the formation of significant anti‐H antibody levels, restricted to the rare *Bombay* type (Oh) individual 76. The plasma of the blood group O(H) individual exerts strong anti‐A/Tn reactive IgM, or anti‐A isoagglutinin activity. In blood group A, the appearance of this ancestral anti A activity is, independently of classic clonal selection, reduced or excluded by human‐specific, A‐allelic GaNAc glycosylation, termed glycosidic exclusion 80 or accommodation, which hypothetically provides the conversion of synthesized glycoconjugates into phenotype‐specific plasma (glyco) proteins and/or molecular complexes that become subject to internalization 82.

The “bulky” GalNAc molecule 37 is a preferred substrate and target of hexosamine epimerization in microorganisms 38 due to undefined biophysical properties, which also dominate the carbohydrate metabolism in mammalian embryonic stem cell‐germ cell (ESC‐GC) transformation. While the role of specific carbohydrates in sperm‐egg recognition remains the subject of discussion 39, 40, 41, the nonsomatic process of GC maturation is initiated by transient *O*‐GalNAc glycosylation 42, 43, which occurs in particular on polypeptides 25 that express trans‐species functional hydrophilic Ser and Thr residues 44. As the most complex and differentially regulated step in protein glycosylation, up to 20 distinct polypeptide *O*‐GalNAc transferases catalyze the first addition of GalNAc to a protein 13, 45, 46. resulting in transient “immature”^42^ *O*‐GalNAc expressions, which are characterized by extremely short half‐lives and identical with Tn antigen formation (Fig. 3). Contrary to a previous report 47, these ancestral, early ontogenetic and genetically undefined functions of “A‐like” *O*‐GalNAc transferases, while used by all metazoan eukaryotes, must be differentiated from species‐intrinsic and human A‐allelic enzyme functions, which are expressed only after formation of the zygote and involve both *N*‐ and *O*‐glycosylations determining phenotype formation based on human‐specific fucosylations (Fig. 4).

Historically, the Tn antigen, or “T nouvelle”, was named upon its discovery in 1957^20^ to emphasize its distinction from the functionally similar T (Thomsen‐Friedenreich) antigen reported in 1930 48, which refers to the disaccharide Gal*β*1‐3GalNAc*α*‐1‐*O*‐Ser/Thr (Fig. 3). Thus, the Tn antigen appears structurally to be less developed than the T antigen; the former predominates in carcinogenesis 49 and is associated with poorer prognosis compared with the latter. Recent reviews have widely discussed and summarized the complex biochemistry of these “A‐like” glycans, their impact on cell differentiation, and their roles in metabolic pathways related to different cancer types and stages, as well as the development of vaccines targeting “A‐like” glycans 50, 51.

In healthy organisms, these cryptic and potentially “aberrant” structures may be specifically reflected by natural anti‐Tn and anti‐T antibodies that are among the anti‐glycan moieties present in the plasma of all mammals 4. In humans, anti‐Tn and anti‐T antibody levels are highly dependent on the ABO(H) blood group and are primarily expressed through their cross‐reactivity with anti‐A/B isoagglutinins 52, 53, 54. Blood group O(H) sera bind to both the Tn and T antigens, whereas blood group A individuals exhibit poor natural anti‐Tn reactivity 55 that in blood group O(H) individuals 56 contributes to elevated anti‐A reactivity. Recent clinical investigations of patients with pancreatic cancer by Hofmann et al. 57 demonstrated that the anti‐A isoagglutinin levels in blood group O(H) and blood group B sera are associated with strong anti‐Tn antibody, which does not react with B or T glycoconjugates. In contrast, the anti‐B antibodies of blood group A sera and O(H) sera bind to B and T glycoconjugates but not to A or Tn glycoconjugates. The authors suggested that this selective cross‐reactivity of isoagglutinins with Tn and T structures is due to their phenotype‐specific terminal moieties; indeed, the terminal N‐acetylgalactosamine is shared by A and Tn antigens, and the terminal galactose is, although with different configuration, shared by B and T antigens (Fig. 3). Friedenreich and Munck had suggested the presence of a potentially authentic anti‐T antibody 48 but to date this antibody has not been confirmed. Thus, in view of the most likely common molecular origin of anti‐A and anti‐Tn reactivity, it is tempting to speculate that the natural anti‐Tn‐reactive IgM and natural human anti‐A isoagglutinin represent a single antibody quality. However, monoclonal anti‐Tn‐specific antibodies have been produced; mice immunized with membrane preparations of human lung samples reacted specifically with the majority of human adenocarcinoma specimens, irrespective of the ABO status of the host, as well as with normal tissues and red cells of blood group A individuals 58. Furthermore, a monoclonal anti‐IgG3 antibody directed against the Tn antigen and not cross‐reactive with the A antigen was generated after mice were immunized with purified Tn antigen 59. A similar immunoglobulin was generated through somatic cell hybridization after mice were immunized with a tumor cell line carrying a Tn‐specific mucin 60. Thus, although the Tn‐ and T‐bearing *O*‐glycans may only represent the metabolic accumulation of short *O*‐glycans, which develop in various cancers for innumerable reasons, these molecules and/or their derivatives clearly show authentic antigenic potential but are potentially synthesized by different *O*‐GalNAc transferase qualities. In view of the more recent experiments by Blixt et al. 61., the chemical simplicity of the Tn antigen does not necessarily stand for an antigenic unity. The authors generated different anti‐Tn monoclonal antibodies of IgM and IgG classes and showed that monoclonal IgM binds to the terminal GalNAc residue of the Tn antigen irrespective of the peptide context and with low selectivity to the glycoproteins, while monoclonal IgG recognizes the Tn antigen in the context of a specific peptide motif. Thus, the Tn antigen‐antibody binding capacity appeared to be determined by the peptide context of the Tn antigen, moreover, antigenic specificity of the antibody and class of the immunoglobulins. Nevertheless, the broad specificity of the “naturally occurring” anti‐A/Tn cross‐reactive IgM molecule most likely covers the major spectrum of antigenic sites and lets distinct anti‐A and Tn reactivities look like a single antibody quality.

Tn‐ and T‐ glycosylation is not restricted to higher metazoan organisms. These *O*‐glycosylations are already used by mollusks 26, and the T antigen appears to be normally expressed on the surface of eggs and liver cells of *Schistosoma mansoni*. Sera from patients infected with this worm produce antibodies against cancerous tissue, whereas experimentally infected mice generate antibodies against Tn and T antigenic epitopes 62. Furthermore, upon their accumulation in vertebrate tissue, invertebrate immune systems recognize Tn and T antigenic epitopes or aberrant “A‐like” structures specifically via two pathways. The egg‐protecting hemagglutinating protein from *H. pomatia* has been established as a tumor cell marker and a prognostic indicator of different human tumor cell lineages 63, and its hexameric structure 64 may give rise to speculation regarding an evolutionary relationship to the mammalian nonimmune or ancestral immunoglobulin M. These molluscan agglutinins are produced in the albumen gland (connected to the oviduct), and emerging from the coat proteins of fertilized eggs. They most likely reflect the snail‐intrinsic, reversible *O*‐GalNAc glycosylations 64, 65, initiating protein glycosylations even in mollusks 26. While the agglutinins are engaged in self‐defense, the agglutinin‐free hemocyanin from *H. pomatia* (HPH) exerted strong anti‐proliferative effects in murine models of colon carcinoma 66. In addition, *Concholepas* hemocyanin inhibits the growth of bladder tumors 67, and the Gal(*β*1‐3)GalNAc‐bearing hemocyanin of *Megathura crenulata* (keyhole limpet hemocyanin, KLH) shows cross‐reactivity with T antigen 68, inducing a potent Th1‐dominant immune response 69 and was used as an effective immunogenic carrier in dendritic cell vaccination developed for immunotherapy of human B cell lymphoma 70.

The metabolic relationship of the Tn and T antigens to other developmental antigens, such as the heterogenetic Forssman antigen, with the structure GalNAc*α*1‐3GalNAc*β*1‐3Gal*α*1‐4Gal*β*1‐4Glc‐R, remains unknown. While Tn and T are common trans‐species, metazoan structures that occur even in mollusks 26, one must differentiate between Forssman‐positive (F+) metazoans, such as mice, and the Forrsman negative (F‐) human. Hakomori et al. described chemically and immunologically detectable levels of the Forssman glycolipid as a normal component of the human gastrointestinal mucosa 71, while they discovered Forssman glycolipids in the tumors of F‐ individuals but did not find them in F+ individuals. Although such F+ tumors arise independently of the ABO(H) blood group, they exert strong cross‐reactivity with blood group A determinants, whereas the Forssman antibody also occurs independently of the ABO(H) blood group 72.

## Nonsomatic trans‐species, A‐like *O*‐GalNAc glycosylations are distinct from somatic species‐intrinsic and blood group A phenotype‐determining GalNAc glycosylations

The above‐described developmental, nonsomatic, and genetically undefined A‐like *O*‐GalNAc transferases are present in any developing metazoan independent of species and phenotype. In fact, these ancestral transferases must be differentiated from strain‐ and species‐intrinsic enzymes, and contrary to a previous report 47, they must be differentiated especially from the human blood group A phenotype‐determining enzyme proteins or functions, as illustrated in Figures 3 and 4.

After generation of the zygote, the complex construction of human ABO(H) phenotypes is accomplished in the Golgi apparatus trans‐cisternae and vesicles through the membrane‐bound, human‐specific, A‐allelic *α*1‐3‐*N*‐acetylgalactosaminyl transferase T2 and B‐allelic *α*1,3‐galactosyl‐transferase, encoded on chromosome 9. This occurs in human‐specific, epistatic cooperation with the fucosyltransferase 1 (FUT1) and 2 (FUT2), encoded by the H and Se genes on chromosome 19. The membrane‐located *N*‐linked glycosylations are associated with soluble enzyme versions, which independently of the secretor status, are involved in identically specific *N‐* and *O*‐linked glycosylations on (muco) epithelial cells and plasma proteins 73, 74, such as clotting factor VIII 33 and vWF 34, carried by A2M 35, 75. It is important to mention, that the dynamic, functional connection between the A2M structure and FVIII/vWF activity is based on both *N*‐ and *O*‐glycosylations 33, while the levels of A2M‐bound ABO(H) blood group reactivity correlate strictly with the phenotype expression on red cell surfaces 34. Consequently, O'Donnell et al. 34 could show that the ABO(H) blood group reactivity associated with A2M carrying vWF, is markedly reduced in plasma from the *Bombay* blood type that lacks ABO(H) epitope synthesis 76. Although blood group ABO(H)‐specific plasma glycoproteins are primarily cellular products, the functionality of soluble plasma glycotransferase is evident in the experiments by Nagai et al. 1978 77, who transferred UDP‐GalNAc to a blood group O red cell surface by means of an enzyme purified from blood group A_1_ plasma, and converted blood O into blood group A in vitro. Furthermore, when A2M is considered an evolutionarily conserved arm of the innate immune system 36, its functional synergism with the structurally related IgM molecule 78 providing Ser/Thr residues 79, might be essential in relation to ontogenetic immunoglobulin modulation that was termed glycosidic exclusion 80 and/or accommodation, and suggests the functions of soluble plasma or serum transferases. According to this concept, which was inspired by a report that natural IgM loses its polyspecificity in undiluted sera 81, the formation of natural anti‐self‐reactive anti‐A/B reactivity is, aside from classic clonal selection of adaptive immunoglobulin production, reduced or excluded by phenotype‐specific glycosylation or accommodation of plasma proteins (Fig. 4). The resulting glycoconjugates may be subject to complex internalization 82, whereas in blood group O(H) individuals, the unaffected anti‐A and Tn‐cross‐reactive IgM remains involved in the internal and external immune defense processes. Finally, the binding of this nonimmune IgM to an antigen might, like a primary immune response, initiate a secondary response and induce the production of anti‐A/Tn‐reactive IgG 49, 56 associated with T and NK cell activation [19, 21, 22], which in the non‐O blood groups hypothetically is affected by glycosidic competition between phenotype and HPA receptor formation occurring on the T and NK cell surfaces.

The human and mouse genomes are described as laying the foundation of genome zoology 83, and although the mouse might be an unsuitable model for the discordance in the ABO(H) phenotype observed in primates 37, 84, the favorable experimental conditions resulting from the anatomy and physiology of the C57BL/10J inbred mouse strain has contributed to the identification of the germline‐encoded origin of an antibody molecule. This antibody is directed against a common trans‐species and human ontogenetic and/or developmental antigen. As a consequence of early ovariectomy, nonsomatic transferase activities during GC maturation might be responsible for synthesizing A‐like trans‐species functional GalNAc‐modified glycans that have been identified on hydrophilic ovarian glycolipids and are transiently expressed by ESCs and/or pluripotent stem cells (SCs). Together with recent advances in SC physiology, these early observations in mice have led to the hypothesis that the developmental “A‐like” *O*‐GalNAc‐determined oligosaccharides and polypeptide precursors of the natural anti‐A “antibody” are conjunctively synthesized and combine *υ*‐gene activation and *O*‐GalNAc‐glycosylation of the immunoglobulin heavy chain at its complementary regions 80. After cell differentiation and/or maturation are completed, these transient “immature” transferase activities are rapidly depleted 42, 43, resulting in downregulation of the developmentally synthesized GalNAc*α*1‐*O*‐Ser/Thr‐R glycan or Tn antigen, and causing the loss of the glycosidic bonds between cell surfaces and complementary proteins. Consequently, the ancestral anti‐A‐reactive IgM, which has developed as an *O*‐linked cell surface molecule, is released into the circulation (Figs. 1 and 4) and displays the respective breaking points, as there are the hydroxy (‐OH) groups of the germline‐specific Ser and/or Thr residues (Fig. 4).

The polyreactivity of the secretory “natural” IgM molecule is assumed to be provided primarily by hydrophilic amino acids. Ser residues, in particular, located on the V regions 79 and assumed to guarantee energy‐rich polyspecificity 85, are appropriate targets for *O*‐GalNAc glycosylation, while the characteristic lack of *O*‐glycans and the presence of Ser/Thr residues on the secretory IgM strongly argue for a “broken linkage” to the developmental “lost” GalNAc*α*1‐*O*‐Ser/Thr‐R glycan or Tn antigen. For example, the presence of *O*‐GalNAc glycan‐bearing glycolipids in differentiating murine ovarian tissue and appearance of the complementary IgM in plasma 14, 15, 16, could represent such “broken linkage”. Enzymes catalyze forward and backward reactions, and in view of the dynamics of *O*‐GalNAc glycosylation 24, 25, 86, 87, the binding of some short *O*‐glycans on cell surfaces and antibody molecules might occur only fleetingly in reversible *O*‐glycosylations 88. Moreover, apart from *N*‐glycosylations, dominating the complex ABO(H) phenotype construction, the hydroxy groups (‐OH) of Ser and Thr residues may serve as predetermined breaking points, on which trans‐species glycans hypothetically are replaced by species‐specific ones in a fast deglycosylation/glycosylation process that may be termed “single cycle event”^89^. In the human blood group O(H), such predetermined breaking points are suggested in the anti‐A‐complementary domain of the IgM molecule and the vis‐à‐vis ABO(H)‐convertible red cell surface 77, on which “lost” ancestral glycans are not replaced by phenotype‐specific ones. While in the phenotype A(H) such replacement has been accomplished and excluded the formation of anti‐self‐reactive IgM, this hypothesis explains the pronounced occurrence of anti‐A and cross‐reactive anti‐Tn in blood group O(H). Clearly, the central immunological position of the human histo (blood) group O(H) 11 is evident in its comprehensive production of both nonimmune and adaptive, environmentally acquired antibodies against all mature A and B glycans involving their cross‐specific developmental glycans, Tn and T, as illustrated in Figure 3. While IgM polyreactivity in the phenotype A(H) individual thus is impaired, the anti‐A/Tn cross‐reactivity in the phenotype O(H) individual potentially contributes to a currently discussed survival advantage 90, 91 in the overall risk of developing cancer when compared with non‐O(H) blood group individuals.

## Conclusions

IgM molecule production *per se* is not restricted to B cells and lymphoid tissues; functional IgM secretion has been demonstrated in normal 1, 2 and malignant human epithelial cells 3, while the formation of immunoglobulins that arise *de novo* from ovarian tissue appears to be established 92. According to Jerne, “*Germ cells of an animal carry a set of v‐genes determining the combining sites of antibodies directed against a complete set of a certain class of histocompatibility antigens of the species to which this animal belongs*” 93. Intriguingly, most ovarian and testicular tumors in humans appear to be B‐cell lymphomas 94, 95 or develop as GC tumors together with non‐Hodgkin lymphoma cells 96, while a primary ovarian tumor has been detected in a single lymph node 97. Moreover, the microenvironment of GC tumors harbors a prominent antigen‐driven humoral response;^98^ thus, these authors speculated that the evolutionary and/or developmental mystery of the relationship between GCs and B lymphocytes might be explained through the molecular biology of B‐cell tumors. However, because the ovary represents the last evolutionary/developmental location in mammals 80, where parthenogenetic potential remains, even in humans 99, 100, such an explanation may also reside in the topographically and molecularly connected synthesis of the trans‐species evolutionary/developmental GalNAc1*α*‐*O*‐Ser/Thr‐R Tn epitope and its authentic complementary protein or nonimmune ancestral IgM molecule occurring in mammalian ovarian tissue. This dynamic connection might be documented by the early experiments of the author, in particular, a timed ovariectomy performed on C57BL/10 mice 14, 15, 16, and in view of the molecular biological data accumulated over the decades in the literature, the 40‐year‐old prediction that the majority of the human isoantibody populations basically reflects growth processes 16 may be substantiated. In fact, the ancestral, innate anti‐A/Tn cross‐reactive IgM dominates these antibody populations and may give rise to speculation of an evolutionary relationship to the hexameric structure 64 of the *O*‐glycan–reactive HPA. This hemagglutinin emerges from the coat proteins of fertilized eggs and most likely reflects the snail–intrinsic, reversible *O*‐GalNAc glycosylations 26, 65, synthesizing the hemocyanins, while all GalNAc expression in *Helix pomatia* and other snails appears to be normal and does not signify malignancy. In these lower metazoans, the fundamental evolutionary missions of reproduction and defense occur topographically and molecularly connected with the function of the albumen gland 101, 102, which produces the multifunctional egg coat proteins that protect the egg against fungal or bacterial attacks. It is intriguing how the female C57BL/10 mouse mimics this developmental connection of reproduction and primitive immunological defense, in which similarly to HPA release from fertilized eggs, the anti‐A/Tn cross‐reactive, nonimmune protein or ancestral IgM is released after completion of CG maturation (Figs. 1 and 2). In humans, these functions are strongly divided topographically and molecularly. Beyond that, in the non‐O blood groups, the physiological anti‐A and cross‐reactive anti‐Tn complementarity of the ancestral IgM molecule undergoes a complex phenotype‐specific enzymatic accommodation 80. It is, aside from clonal selection, primarily this human phenotype‐specific, glycosidic accommodation of plasma proteins that clearly affects the natural IgM polyreactivity, and the reduction of physiological anti‐self‐reactivity potentially increases the risk of developing “aberrant” structures and/or cancerous tissue, which might be the price of species specializing and phenotype diversity.

## Conflict of Interest

None declared.

## References

[1] Zhou, R. , S. P. O'Hara , and X. M. Chen . 2011 MicroRNA regulation of innate immune responses in epithelial cells. Cell. Mol. Immunol. 8:371–379. 21725335 10.1038/cmi.2011.19 PMC4012889

[2] Shao, W. , F. Hu , J. Ma , C. Zhang , Q. Liao , Z. Zhu , et al. 2016 Epithelial cells are a source of natural IgM that contribute to innate immune responses. Int. J. Biochem. Cell Biol. 73:19–29. 26820901 10.1016/j.biocel.2016.01.017

[3] Hu, F. , L. Zhang , J. Zheng , et al. 2012 Spontaneous production of immunoglobulin M in human epithelial cancer cells. PLoS ONE 7 https://doi.org/10.1371/journal.pone.0051423. 10.1371/journal.pone.0051423 PMC3520907 23251529

[4] Muthana, S. , and J. Gildersleeve . 2016 Factors affecting Anti‐Glycan IgG and IgM repertoires in human serum. Sci. Rep. 6:19509. 26781493 10.1038/srep19509 PMC4726023

[5] Springer, G. , H. Horton , and M. Forbes . 1959 Origin of anti‐human blood group B agglutinins in white Leghorn chicks. J. Exp. Med. 110:221–244. 13673136 10.1084/jem.110.2.221 PMC2136992

[6] Springer, G. F. , P. Williamson , and W. C. Brandes . 1961 Blood group activity of gram‐negative bacteria. J. Exp. Med. 113:1077–1093. http://www.pubmedcentral.nih.gov/articlerender.fcgi?artid=2137423&tool=pmcentrez&rendertype=abstract. 19867191 10.1084/jem.113.6.1077 PMC2137423

[7] Springer, F. , and R. Horton . 1969 Blood group isoantibody stimulation in man by feeding blood group‐active bacteria. J. Clin. Invest. 48:1280–1291. 4893685 10.1172/JCI106094 PMC322351

[8] Arend, P. , and G. Fehlhaber . 1969 Varying influence of increased enteral antigen absorption on the behavior of “natural” antibodies in O and A blood group subjects. Comparative blood group serological studies on patients with ulcerative colitis and healthy persons [Article in German]. J. Mol. Med. (Klinische Wochenschrift) 47:535–541. 10.1007/BF01715818 5382535

[9] O'Keefe, D. S. , and A. Dobrovic . 1996 A rapid and reliable PCR method for genotyping the ABO blood group. II: A2 and O2 alleles. Hum Mutat. 8:358–361. Available from: http://www.ncbi.nlm.nih.gov/pubmed/8956041. 8956041 10.1002/(SICI)1098-1004(1996)8:4<358::AID-HUMU9>3.0.CO;2-3

[10] Yazer, M. H. , B. Hosseini‐Maaf , and M. L. Olsson . 2008 Blood grouping discrepancies between ABO genotype and phenotype caused by O alleles. Curr Opin Hematol. 15:618–624. 18832934 10.1097/MOH.0b013e3283127062

[11] Arend, P . 2017 Central immunological position of the human histo (blood) group O(H). https://doi.org/10.6084/m9.figshare.4714618.v91.

[12] Chuang, J. , C. Hung , S. Chang , T. Chou , and P. Lee . 2008 Does Immunosuppressive Pharmacotherapy Affect Isoagglutinin Titers?. Transplant. Proc. 40:2685–2687. 18929836 10.1016/j.transproceed.2008.08.018

[13] Bennett, E. , U. Mandel , H. Clausen , T. Gerken , T. Fritz , and L. Tabak . 2012 Control of mucin‐type O‐glycosylation: A classification of the polypeptide GalNAc‐transferase gene family. Glycobiology 22:736–756. 22183981 10.1093/glycob/cwr182 PMC3409716

[14] Arend, P. , and J. Nijssen . 1976 Significance of specific ovarian receptors for syngeneic naturally‐occurring haemagglutinating anti‐A antibodies. Immunogenetics 3:373–382. 10.1111/j.1744-313x.1976.tb00598.x 1010947

[15] Arend, P. , and J. Nijssen . 1977 Age‐dependent appearance of A‐specific ovarian glycolipids and syngeneic “natural” anti‐A hemolysin in mice. Z. Immunitatsforsch. Immunobiol. 153:74–84. 868207

[16] Arend, P. , and J. Nissen . 1977 A‐specific autoantigenic ovarian glycolipids inducing production of “natural” anti‐A antibody. Nature 269:255–257. 563514 10.1038/269255a0

[17] Arend, P. 1971 Observations on different origins of “naturally‐occurring” antibodies. Eur. J. Immunol. 1:398–402. 5157762 10.1002/eji.1830010520

[18] Arend, P. 1980 An auto‐reactive A‐like ovarian determinant distinct from xeno‐reactive A‐like structures. Immunobiology 156:410–417. 6154644 10.1016/S0171-2985(80)80074-X

[19] Axelsson, B. , A. Kimura , S. Hammarström , H. Wigzell , K. Nilsson , and H. Mellstedt . 1978 Helix pomatia A hemagglutinin: selectivity of binding to lymphocyte surface glycoproteins on T cells and certain B cells. Eur. J. Immunol. 8:757–764. 309819 10.1002/eji.1830081102

[20] Moreau, R. , J. Dausset , J. Bernard , and J. Moullec . 1957 Acquired hemolytic anemia with polyagglutinability of erythrocytes by a new factor present in normal blood (Article in French). Bull. Mem. Soc. Med. Hop. Paris. 73:569–587. 13460699

[21] Haller, O. , M. Gidlund , U. Hellström , S. Hammarström , and H. Wigzell . 1978 A new surface marker on mouse natural killer cells: receptors for Helix pomatia A hemagglutinin. Eur. J. Immunol. 8:765–771. 309820 10.1002/eji.1830081103

[22] Poros, A. , L. Ahrlund‐Richter , E. Klein , S. Hammarström , and N. Koide . 1983 Expression of Helix pomatia (HP) haemagglutinin receptors on cytolytic lymphocytes activated in mixed cultures. J. Immunol. Methods 57:9–19. 6600771 10.1016/0022-1759(83)90059-5

[23] van Vliet, S. J. , I. M. Vuist , K. Lenos , B. Tefsen , H. Kalay , J. J. García‐Vallejo , et al. 2013 Human T cell activation results in extracellular signal‐regulated kinase (ERK)‐calcineurin‐dependent exposure of Tn antigen on the cell surface and binding of the macrophage galactose‐type lectin (MGL). J. Biol. Chem. 288:27519–27532. 23918927 10.1074/jbc.M113.471045 PMC3779747

[24] Steentoft, C. , S. Y. Vakhrushev , H. J. Joshi , et al. 2013 Precision mapping of the human O‐GalNAc glycoproteome through simplecell technology. EMBO J. 32:1478–1488. 23584533 10.1038/emboj.2013.79 PMC3655468

[25] Brockhausen, I. , H. Schachter , and P. Stanley . 2009 O‐GalNAc Glycans Pp. 1–16 *in* Varki A., Cummings R. D., Esko J. D., Freeze H. H., Stanley P., Bertozzi C. R., Hart G. W., Etzler M. E., eds. Source Essentials of Glycobiology. 2nd ed.Cold Spring Harbor (NY): Cold Spring Harbor Laboratory Press. Chapter 9. 20301232

[26] Staudacher, E. 2015 Mucin‐Type O‐Glycosylation in Invertebrates. Molecules 20:10622–10640. 26065637 10.3390/molecules200610622 PMC6272458

[27] Peiris, D. , M. Ossondo , S. Fry , M. Loizidou , J. Smith‐Ravin , and M. Dwek . 2015 Identification of O‐linked glycoproteins binding to the lectin Helix pomatia agglutinin as markers of metastatic colorectal cancer. PLoS ONE 10 https://doi.org/10.1371/journal.pone.0138345. 10.1371/journal.pone.0138345 PMC4619703 26495974

[28] Welinder, C. , B. Jansson , M. Ferno , H. Olsson , and B. Baldetorp . 2009 Expression of Helix pomatia lectin binding glycoproteins in women with breast cancer in relationship to their blood group phenotypes. J. Proteome Res. 8:782–787. 18998722 10.1021/pr800444b

[29] Laack, E. , H. Nikbakht , A. Peters , C. Kugler , Y. Jasiewicz , L. Edler , et al. 2002 Lectin histochemistry of resected adenocarcinoma of the lung: helix pomatia agglutinin binding is an independent prognostic factor. Am. J. Pathol. 160:1001–1008. 11891197 10.1016/S0002-9440(10)64921-8 PMC1867178

[30] Thies, A. , I. Moll , J. Berger , and U. Schumacher . 2001 Lectin binding to cutaneous malignant melanoma: HPA is associated with metastasis formation. Br. J. Cancer 84:819–823. 11259098 10.1054/bjoc.2000.1673 PMC2363810

[31] Hellström, U. , S. Hammarström , and G. Klein . 1978 Enrichment of Helix pomatia (HP) lectin binding variant from the TA3St mouse ascites tumor by repeated column selection. Eur. J. Cancer 14:1665–1772. 10.1016/0014-2964(78)90234-7 738331

[32] Schreiber, S. , A. Gocht , F. Wegwitz , W. Deppert , and U. Schumacher . 2014 Lectin histochemistry of murine WAP‐T mammary cancer reveals similar glycoconjugate changes to those in human breast cancer. Anticancer Res. 34:7045–7053. 25503131

[33] O'Sullivan, J. M. , P. V. Jenkins , O. Rawley , et al. 2016 Galectin‐1 and galectin‐3 constitute novel‐binding partners for Factor VIII. Arterioscler. Thromb. Vasc. Biol. 36:855–863. 27013611 10.1161/ATVBAHA.115.306915

[34] O'Donnell, J. S. , T. A. J. McKinnon , J. T. B. Crawley , D. A. Lane , and M. A. Laffan . 2005 Bombay phenotype is associated with reduced plasma‐VWF levels and an increased susceptibility to ADAMTS13 proteolysis. Blood 106:1988–1991. 15886321 10.1182/blood-2005-02-0792

[35] Matsui, T. , J. Hamako , Y. Ozeki , and K. Titani . 2001 Comparative study of blood group‐recognizing lectins toward ABO blood group antigens on neoglycoproteins, glycoproteins and complex‐type oligosaccharides. Biochim Biophys Acta ‐ Gen Subj. 1525:50–57. 10.1016/s0304-4165(00)00170-7 11342253

[36] Armstrong, P. B. , and J. P. Quigley . 1999 Alpha2‐macroglobulin: an evolutionarily conserved arm of the innate immune system. Dev. Comp. Immunol. 23:375–390. 10426429 10.1016/s0145-305x(99)00018-x

[37] Yamamoto, M. , X. Lin , Y. Kominato , Y. Hata , R. Noda , N. Saitou , et al. 2001 Murine equivalent of the human histo‐blood group ABO Gene is a cis‐AB Gene and encodes a glycosyltransferase with both a and b transferase activity. J. Biol. Chem. 276:13701–13708. 11278752 10.1074/jbc.M010805200

[38] Cunneen, M. , B. Liu , L. Wang , and P. Reeves . 2013 Biosynthesis of UDP‐GlcNAc, UndPP‐GlcNAc and UDP‐GlcNAcA involves three easily distinguished 4‐Epimerase Enzymes, Gne, Gnu and GnaB. PLoS ONE 8 https://doi.org/dx.doi.org/10.1371/journal.pone.0067646. 10.1371/journal.pone.0067646 PMC3682973 23799153

[39] Clark, G. F. 2011 Molecular models for mouse sperm‐oocyte binding. Glycobiology 21:3–5. 21188842 10.1093/glycob/cwq159

[40] Bianchi, E. , B. Doe , D. Goulding , and G. J. Wright and Sanger Mouse Genetics Project 2 . 2014 Juno is the egg Izumo receptor and is essential for mammalian fertilisation. Nature 508:483–487. 24739963 10.1038/nature13203 PMC3998876

[41] Aydin, H. , A. Sultana , S. Li , A. Thavalingam , and J. Lee . 2016 Molecular architecture of the human sperm IZUMO1 and egg JUNO fertilization complex. Nature 16:562–565. 10.1038/nature18595 PMC5319863 27309818

[42] Reisner, Y. , L. Itzicovitch , A. Meshorer , and N. Sharon . 1978 Hemopoietic stem cell transplantation using mouse bone marrow and spleen cells fractionated by lectins. Proc. Natl Acad. Sci. USA 75:2933–2936. 26916 10.1073/pnas.75.6.2933 PMC392680

[43] Nash, R. , L. Neves , R. Faast , M. Pierce , and S. Dalton . 2007 The Lectin dolichos biflorus agglutinin recognizes glycan epitopes on the surface of murine embryonic stem cells: a new tool for characterizing pluripotent cells and early differentiation. Stem Cells 25:974–982. 17170066 10.1634/stemcells.2006-0224

[44] Saini, S. , N. Maiti , and A. Kaushik . 2013 Partial characterization of immunoglobulin C gene of water buffalo (Bubalus bubalis) predicts distinct structural features of C1q‐binding site in C 3 domain. Int J Microbiol Adv Immunol. 1:19–23. doi: dx.doi.org/10.19070/2329‐9967‐130004.

[45] Tenno, M. , K. Ohtsubo , F. Hagen , D. Ditto , A. Zarbock , P. Schaerli , et al. 2007 Initiation of protein O glycosylation by the polypeptide GalNAcT‐1 in vascular biology and humoral immunity. Mol. Cell. Biol. 27:8783–8796. 17923703 10.1128/MCB.01204-07 PMC2169402

[46] Schjoldager, K. , and H. Clausen . 2012 Site‐specific protein O‐glycosylation modulates proprotein processing ‐ Deciphering specific functions of the large polypeptide GalNAc‐transferase gene family. Biochim Biophys Acta ‐ Gen Subj. 1820:2079–2094. 10.1016/j.bbagen.2012.09.014 23022508

[47] Arend, P .2014 Complementary innate (anti‐A‐specific) IgM emerging from ontogenic O‐GalNAc‐transferase depletion (Innate IgM complementarity residing in ancestral antigen completeness). Immunobiology 219:285–295. www.elsevier.com/locate/imbio. 24290972 10.1016/j.imbio.2013.10.011

[48] Friedenreich, V. , and J. Munck . 1930 Intravital Effects of „Transformed” Blood Corpuscels in Guinea‐Pigs. Acta Pathol. Microbiol. Scand. 7:134–145.

[49] Springer, G. F. 1997 Immunoreactive T and Tn epitopes in cancer diagnosis, prognosis, and immunotherapy. J. Mol. Med. 75:594–602. 9297627 10.1007/s001090050144

[50] Ju, T. , V. I. Otto , and R. D. Cummings . 2011 The Tn antigen‐structural simplicity and biological complexity. Angew Chemie Int Ed English. 50:1770–1791. 10.1002/anie.201002313 PMC7159538 21259410

[51] Chia, J. , G. Goh , and F. Bard . 2016 Short O‐GalNAc glycans: regulation and role in tumor development and clinical perspectives. Biochim. Biophys. Acta. 1860:1623–1639. https://doi.org/10.1016/j.bbagen.2016.03.008. 26968459 10.1016/j.bbagen.2016.03.008

[52] Dahr, W. , G. Uhlenbruck , H. Gunson , and M. Van Der Hart . 1975 Molecular basis of Tn‐polyagglutinability. Vox Sang. 29:36–50. 1173700 10.1111/j.1423-0410.1975.tb00475.x

[53] Rögener, W. , L. Renwrantz , and G. Uhlenbruck . 1986 Comparison of a hemolymph lectin from Octopus vulgaris with hemocyanin. Comp. Biochem. Physiol. – Part B Biochem. 85:119–123.

[54] Bird, G. , N. Shinton , and J. Wingham . 1971 Persistent mixed‐field polyagglutination. Br. J. Haematol. 21:443–453. 5122668 10.1111/j.1365-2141.1971.tb02705.x

[55] Jaff, M. 2010 Higher frequency of secretor phenotype in O blood group – its benefits in prevention and/or treatment of some diseases. Int. J. Nanomedicine. 5:901–905. https://doi.org/10.2147%2FIJN.S13980. 21116330 10.2147/IJN.S13980 PMC2990383

[56] Smorodin, E. , O. Kurtenkov , B. Sergeyev , A. Lilleorg , and V. Chuzmarov . 2001 Antibodies to tumor‐associated epitopes in sera of cancer patients and blood donors. Exp. Onkol. 23:109–113.

[57] Hofmann, B. T. , A. Stehr , T. Dohrmann , C. Güngör , L. Herich , J. Hiller , et al. 2014 ABO Blood Group IgM isoagglutinins interact with tumor‐associated O‐glycan structures in pancreatic cancer. Clin. Cancer Res. 20:6117–6126. 25320359 10.1158/1078-0432.CCR-14-0716

[58] Hirohashi, S. , H. Clausen , T. Yamada , Y. Shimosato , and S. Hakomori . 1985 Blood Group A cross‐reacting epitope defined by monoclonal antibodies NCC‐LU‐35 and ‐81 expressed in cancer of blood group O or B individuals: its identification as Tn antigen. Proc. Natl Acad. Sci. 82:7039. 2413456 10.1073/pnas.82.20.7039 PMC391305

[59] Takahashi, H. , R. Metoki , and S. Hakomori . 1988 Immunoglobulin G3 monoclonal antibody directed to Tn antigen (tumor‐associated *α*‐N‐acetylgalactosaminyl epitope) that does not cross‐react with blood group A antigen. Cancer Res. 48:4361–4367. 3390832

[60] Welinder, C. , B. Baldetorp , C. Borrebaeck , B. Fredlund , and B. Jansson . 2011 A new murine IgG1 anti‐Tn monoclonal antibody with in vivo anti‐tumor activity. Glycobiology 21:1097–1107. 21470982 10.1093/glycob/cwr048

[61] Blixt, K. , O. Lavrova , D. Mazurov , E. Cló , K. Stjepan , N. Bovin , et al. 2012 Analysis of Tn antigenicity with a panel of new IgM and IgG1 monoclonal antibodies raised against leukemic cells. Glycobiology 212AD 22:529–542. 22143985 10.1093/glycob/cwr178 PMC3287017

[62] Thors, C. , B. Jansson , H. Helin , and E. Linder . 2006 Thomsen‐friedenreich oncofetal antigen in schistosoma mansoni : localization and immunogenicity in experimental mouse infection. Parasitology 132(Pt 1):73–81. 16393356 10.1017/S003118200500867X

[63] Brooks, C. , A. Schietinger , S. Borisova , P. Kufer , M. Okon , T. Hirama , et al. 2010 Antibody recognition of a unique tumor‐specific\rglycopeptide antigen. Proc. Natl Acad. Sci. USA 107:10056–11006. 20479270 10.1073/pnas.0915176107 PMC2890472

[64] Sanchez, J. F. , J. Lescar , V. Chazalet , A. Audfray , J. Gagnon , R. Alvarez , et al. 2006 Biochemical and structural analysis of Helix pomatia agglutinin: a hexameric lectin with a novel fold. J. Biol. Chem. 281:20171–20180. 16704980 10.1074/jbc.M603452200

[65] Stepan, H. , M. Pabst , F. Altmann , H. Geyer , R. Geyer , and E. Staudacher . 2012 O‐Glycosylation of snails. Glycoconj. J. 29:189–198. 22581130 10.1007/s10719-012-9391-4 PMC3372779

[66] Gesheva, V. , S. Chausheva , N. Mihaylova , I. Manoylov , L. Doumanova , K. Idakieva , et al. 2014 Anti‐cancer properties of gastropodan hemocyanins in murine model of colon carcinoma. BMC Immunol. 15:34. 25168124 10.1186/s12865-014-0034-3 PMC4164791

[67] Moltedo, B. , F. Faunes , D. Haussmann , P. De Ioannes , A. De Ioannes , J. Puente , et al. 2006 Immunotherapeutic effect of concholepas hemocyanin in the murine bladder cancer model: evidence for conserved antitumor properties among hemocyanins. J. Urol. 176:2690–2695. 17085197 10.1016/j.juro.2006.07.136

[68] Wirguin, I. , L. Suturkova‐Milosevic , C. Briani , and N. Latov . 1995 Keyhole limpet hemocyanin contains Gal(beta 1‐3)‐GalNAc determinants that are cross‐reactive with the T antigen. Cancer Immunol. Immunother. 40:307–310. 7600562 10.1007/BF01519630 PMC11037588

[69] Zhong, T. , S. Arancibia , R. Born , R. Tampe , J. Villar , M. Del Campo , et al. 2016 Hemocyanins stimulate innate immunity by inducing different temporal patterns of proinflammatory cytokine expression in macrophages. J. Immunol. 196:4650–4662. 27183578 10.4049/jimmunol.1501156 PMC4873724

[70] Timmerman, J. , D. Czerwinski , T. Davis , F. Hsu , C. Benike , Z. Hao , et al. 2002 Idiotype‐pulsed dendritic cell vaccination for B‐cell lymphoma: clinical and immune responses in 35 patients. Blood. 99:1517–1526. 11861263 10.1182/blood.v99.5.1517

[71] Hakomori, S. , S. M. Wang , and W. W. Young . 1977 Isoantigenic expression of Forssman glycolipid in human gastric and colonic mucosa: its possible identity with “A‐like antigen” in human cancer. Proc. Natl Acad. Sci. USA 74:3023–3027. 268649 10.1073/pnas.74.7.3023 PMC431392

[72] Kijimoto‐Ochiai, S. , W. Takahashi , and A. Makita . 1981 Anti‐Forssman antibody in human sera: properties and decreased level in cancer patients. Jpn. J. Exp. Med. 51:149–155. 7300030

[73] Preston, R. , O. Rawley , E. Gleeson , and J. O'Donnell . 2013 Elucidating the role of carbohydrate determinants in regulating hemostasis: insights and opportunities. Blood 1212:3801–3810. 10.1182/blood-2012-10-415000 23426946

[74] Holgersson, J. , L. Jining , L. Lindberg , and P. Grufman . 2013 Blood group antigens of different types for diagnostic and therapeutic applications. US 8404456 B2.

[75] Matsui, T. , Y. Fujimura , S. Nishida , and K. Titani . 1993 Human plasma alpha 2‐macroglobulin and von Willebrand factor possess covalently linked ABO(H) blood group antigens in subjects with corresponding ABO phenotype. Blood 82:663–668. 7687165

[76] Bhende, Y. M. , C. K. Deshpande , H. M. Bhatia , R. Sanger , R. R. Race , W. T. Morgan , et al. 2008 A “new” blood‐group character related to the ABO system. 1952. Natl Med. J. India 21:3 p. 19320334

[77] Nagai, M. , V. Davè , B. Kaplan , and A. Yoshida . 1978 Human blood group glycosyltransferases. I. Purification of n‐acetylgalactosaminyltransferase. J. Biol. Chem. 253:377–379. 618875

[78] Stevenson, L. , E. Laursen , G. J. Cowan , B. Bandoh , L. Barfod , D. R. Cavanagh , et al. 2015 *α*2‐Macroglobulin can crosslink multiple plasmodium falciparum erythrocyte membrane protein 1 (PfEMP1) molecules and may facilitate adhesion of parasitized erythrocytes. PLoS Pathog. 11:e1005022. 26134405 10.1371/journal.ppat.1005022 PMC4489720

[79] Wang, H. , J. E. Coligan , and H. C. Morse . 2016 Emerging functions of natural IgM and its Fc receptor FCMR in immune homeostasis. Front Immunol. 7:99 https://doi.org/10.3389/fimmu.2016.00099PMID: 27014278. 27014278 10.3389/fimmu.2016.00099 PMC4791374

[80] Arend, P. 2016 ABO (histo) blood group phenotype development and human reproduction as they relate to ancestral IgM formation: a hypothesis. Immunobiology 221:116–127. 26433867 10.1016/j.imbio.2015.07.003

[81] Chu, Q. , J. J. Ludtke , V. M. Subbotin , A. Blockhin , and A. V. Sokoloff . 2008 The acquisition of narrow binding specificity by polyspecific natural IgM antibodies in a semi‐physiological environment. Mol. Immunol. 45:1501–1513. 17983656 10.1016/j.molimm.2007.07.043 PMC2359650

[82] Gill, D. J. , H. Clausen , and F. Bard . 2011 Location, location, location: new insights into O‐GalNAc protein glycosylation. Trends Cell Biol. 21:149–158. 21145746 10.1016/j.tcb.2010.11.004

[83] Emes, R. , L. Goodstadt , E. Winter , and C. Ponting . 2003 Comparison of the genomes of human and mouse lays the foundation of genome zoology. Hum. Mol. Genet. 12:701–709. 12651866 10.1093/hmg/ddg078

[84] Larkin, J. , and C. Porter . 2005 Mice are unsuitable for modelling ABO discordance despite strain‐specific A cross‐reactive natural IgM. Br. J. Haematol. 130:310–317. 16029461 10.1111/j.1365-2141.2005.05609.x

[85] Willis, J. R. , B. S. Briney , S. L. DeLuca , J. E. Jr. Crowe , and J. Meiler . 2013 Human Germline Antibody Gene Segments Encode Polyspecific Antibodies. PLoS Comput Biol 9:e1003045 https://doi.org/10.1371/journal.pcbi.1003045. 23637590 10.1371/journal.pcbi.1003045 PMC3636087

[86] Agarwal, K. , R. Kaul , M. Garg , A. Shajahan , S. K. Jha , and S. G. Sampathkumar . 2013 Inhibition of Mucin‐type O‐glycosylation through metabolic processing and incorporation of N‐thioglycolyl‐d‐galactosamine peracetate (Ac 5GalNTGc). J. Am. Chem. Soc. 135:14189–14197. 23987472 10.1021/ja405189k

[87] Chia, J. , K. M. Tham , D. J. Gill , E. A. Bard‐Chapeau , and F. A. Bard . 2014 ERK8 is a negative regulator of O‐GalNAc glycosylation and cell migration. Elife 189:843 https://doi.org/10.7554/elife.01828. 10.7554/eLife.01828 PMC3945522 24618899

[88] Lodish, H. , A. Berk , S. Zipursky , P. Matsudaira , D. Baltimore , and J. Darnell . 2001 Biochemistry and Molecular Biology Education, 29:126–128. Freeman & Co., New York, NY, 2000, 1084 pp. https://doi.org/10.1016/s1470-8175(01)00023-6.

[89] Angeletti, R. H . 2012 Proteins: Analysis and Design Pp. 363 *in* Angeletti R. H., ed. Proteins: Analysis and Design. Academic Press, San Diego, London, New York, Sydney, Boston, Tokyo, Toronto, ISBN‐10: 0123885418 ‐ ISBN‐13: 978‐0123885418.

[90] Hsiao, L. , N. Liu , S. You , and L. Hwang . 2015 ABO blood group and the risk of cancer among middle‐aged people in Taiwan. Asia. Pac. J. Clin. Oncol. 11:e31–e36. 25244548 10.1111/ajco.12253

[91] Zhang, B. , N. He , Y. Huang , F. Song , and K. Chen . 2014 ABO blood groups and risk of cancer: a systematic review and meta‐analysis. Asian Pac. J. Cancer Prev. 15:4643–4650. 24969898 10.7314/apjcp.2014.15.11.4643

[92] Hoek, A. , J. Schoemaker , and H. A. Drexhage . 1997 Premature Ovarian Failure and Ovarian Autoimmunity 1. Endocr. Rev. 18:107–134. 9034788 10.1210/edrv.18.1.0291

[93] Jerne, N. K. 1971 The somatic generation of immune recognition. Eur. J. Immunol. 1:1–9. 14978855 10.1002/eji.1830010102

[94] Sun, J. , J. Zhang , Q. Ling , Y. Luo , S. Wu , Z. Liang , et al. 2015 Primary diffuse large B‐cell lymphoma of the ovary is of a germinal centre B‐cell‐like phenotype. Virchows Arch. 466:93–100. 25403088 10.1007/s00428-014-1682-7

[95] Valli, R. , E. Froio , deAlvarez Celis M. , V. Mandato , and S. Piana .2014 Diffuse large B‐cell lymphoma occurring in an ovarian cystic teratoma: expanding the spectrum of large B‐cell lymphoma associated with chronic inflammation. Hum. Pathol. 45:2507–2511. 25439346 10.1016/j.humpath.2014.09.002

[96] Valdez, R. , P. McKeever , W. Finn , S. Gebarski , and B. Schnitzer . 2002 Composite germ cell tumor and B‐cell non‐Hodgkin's lymphoma arising in the sella turcica. Hum. Pathol. 33:1044–1047. 12395379 10.1053/hupa.2002.128063

[97] Carrabin, N. , I. Treilleux , P. Meeus , O. Tredan , and I. Ray‐Coquard . 2013 Primary ovarian borderline tumor in the inguinal lymph node. Int. J. Gynecol. Pathol. 32:167–170. 23370643 10.1097/PGP.0b013e318257def6

[98] Willis, S. , S. Mallozzi , S. Rodig , K. Cronk , S. McArdel , T. Caron , et al. 2009 The microenvironment of germ cell tumors harbors a prominent antigen‐driven humoral response. J. Immunol. 182:3310–3317. 19234230 10.4049/jimmunol.0803424

[99] Kim, K. , K. Ng , P. Rugg‐Gunn , J. Shieh , O. Kirak , R. Jaenisch , et al. 2007 Recombination signatures distinguish embryonic stem cells derived by parthenogenesis and somatic cell nuclear transfer. Cell Stem Cell 1:346–352. 18371368 10.1016/j.stem.2007.07.001

[100] Polak de Fried, E. , P. Ross , G. Zang , A. Divita , K. Cunniff , F. Denaday . et al. 2008 Human parthenogenetic blastocysts derived from noninseminated cryopreserved human oocytes. Fertil. Steril. 89:943–947. 17706204 10.1016/j.fertnstert.2007.04.045

[101] Mullainadhan, P. , and L. Renwrantz . 1989 Comparative analysis of agglutinins from hemolymph and albumin gland of Helix pomatia. J. Comp. Physiol. B. 159:443–452. 2808854 10.1007/BF00692416

[102] Ishiyama, I. , W. Dietz , and G. Uhlenbruck . 1973 Comparative studies of anti‐a agglutinins from various snails of the genus helix (Helix pomatia and Helix aspersa). Comp. Biochem. Physiol – Part B Biochem. 44:529–547. https://doi.org/10.1016/0305-0491(73)90027-8. 10.1016/0305-0491(73)90027-8 4196846

